# Tranexamic Acid for Intracerebral Hemorrhage in Patients on Non-Vitamin K Antagonist Oral Anticoagulants (TICH-NOAC): A Multicenter, Randomized, Placebo-Controlled, Phase 2 Trial

**DOI:** 10.1161/STROKEAHA.123.042866

**Published:** 2023-07-19

**Authors:** Alexandros A. Polymeris, Grzegorz M. Karwacki, Bernhard M. Siepen, Sabine Schaedelin, Dimitrios A. Tsakiris, Christoph Stippich, Raphael Guzman, Christian H. Nickel, Nikola Sprigg, Georg Kägi, Jochen Vehoff, Filip Barinka, Sebastian Thilemann, Marina Maurer, Benjamin Wagner, Christopher Traenka, Henrik Gensicke, Gian Marco De Marchis, Leo H. Bonati, Urs Fischer, Werner J. Z’Graggen, Krassen Nedeltchev, Susanne Wegener, Philipp Baumgartner, Stefan T. Engelter, David J. Seiffge, Nils Peters, Philippe A. Lyrer

**Affiliations:** 1 Department of Neurology and Stroke Center (A.A.P., S.T., M.M., B.W., C.T., H.G., G.M.D.M., L.H.B., U.F., S.T.E., D.J.S., N.P., P.A.L.), University Hospital Basel and University of Basel, Switzerland.; 2 Department of Clinical Research (S.S.), University Hospital Basel and University of Basel, Switzerland.; 3 Department of Neurosurgery (R.G.), University Hospital Basel and University of Basel, Switzerland.; 4 Department of Emergency Medicine (C.H.N.), University Hospital Basel and University of Basel, Switzerland.; 5 Department of Radiology and Nuclear Medicine, Luzerner Kantonsspital, Switzerland (G.M.K.).; 6 Department of Neurology, Inselspital, University Hospital and University of Bern, Switzerland (B.M.S., G.K., U.F., W.J.Z., D.J.S.).; 7 Graduate School of Health Sciences, University of Bern, Switzerland (B.M.S.).; 8 Division of Hematology, University Hospital Basel, Switzerland (D.A.T.).; 9 Department of Neuroradiology and Radiology, Kliniken Schmieder, Allensbach, Germany (C.S.).; 10 Nottingham Stroke Trials Unit, University of Nottingham, United Kingdom (N.S.).; 11 Department of Neurology and Stroke Center, Cantonal Hospital St. Gallen, Switzerland (G.K., J.V.).; 12 Stroke Center, Klinik Hirslanden Zurich, Switzerland (F.B., N.P.).; 13 Neurology and Neurorehabilitation, University Department of Geriatric Medicine Felix Platter, University of Basel, Switzerland (C.T., H.G., S.T.E., N.P.).; 14 Reha Rheinfelden, Switzerland (L.H.B.).; 15 Department of Neurology and Stroke Center, Cantonal Hospital Aarau, Switzerland (K.N.).; 16 Department of Neurology, University Hospital and University of Zurich, Switzerland (S.W., P.B.).; Cantonal Hospital Aarau; Cantonal Hospital Aarau; University Hospital Basel; University Hospital Basel; University Hospital Basel; University Hospital Basel; University Hospital Basel; University Hospital Basel; University Hospital Basel; University Hospital Basel; University Hospital Bern; University Hospital Bern; University Hospital Bern; University Hospital Bern; University Hospital Bern; University Hospital Bern; University Hospital Bern; University Hospital Bern; Cantonal Hospital St. Gallen; Cantonal Hospital St. Gallen; Cantonal Hospital St. Gallen; Cantonal Hospital St. Gallen; Cantonal Hospital St. Gallen; Cantonal Hospital St. Gallen; Klinic Hirslanden Zurich; Klinic Hirslanden Zurich; Klinic Hirslanden Zurich; Klinic Hirslanden Zurich; Klinic Hirslanden Zurich; University Hospital Zurich; University Hospital Zurich

**Keywords:** anticoagulants, cerebral hemorrhage, factor Xa inhibitors, tranexamic acid

## Abstract

**BACKGROUND:**

Evidence-based hemostatic treatment for intracerebral hemorrhage (ICH) associated with non–vitamin K antagonist oral anticoagulants (NOACs) is lacking. Tranexamic acid (TXA) is an antifibrinolytic drug potentially limiting hematoma expansion. We aimed to assess the efficacy and safety of TXA in NOAC-ICH.

**METHODS:**

We performed a double-blind, randomized, placebo-controlled trial at 6 Swiss stroke centers. Patients with NOAC-ICH within 12 hours of symptom onset and 48 hours of last NOAC intake were randomized (1:1) to receive either intravenous TXA (1 g over 10 minutes followed by 1 g over 8 hours) or matching placebo in addition to standard medical care via a centralized Web-based procedure with minimization on key prognostic factors. All participants and investigators were masked to treatment allocation. Primary outcome was hematoma expansion, defined as ≥33% relative or ≥6 mL absolute volume increase at 24 hours and analyzed using logistic regression adjusted for baseline hematoma volume on an intention-to-treat basis.

**RESULTS:**

Between December 12, 2016, and September 30, 2021, we randomized 63 patients (median age, 82 years [interquartile range, 76–86]; 40% women; median hematoma volume, 11.5 [4.8–27.4] mL) of the 109 intended sample size before premature trial discontinuation due to exhausted funding. The primary outcome did not differ between TXA (n=32) and placebo (n=31) arms (12 [38%] versus 14 [45%]; adjusted odds ratio, 0.63 [95% CI, 0.22–1.82]; *P*=0.40). There was a signal for interaction with onset-to-treatment time (*P*_interaction_=0.024), favoring TXA when administered within 6 hours of symptom onset. Between the TXA and placebo arms, the proportion of participants who died (15 [47%] versus 13 [42%]; adjusted odds ratio, 1.07 [0.37–3.04]; *P*=0.91) or had major thromboembolic complications within 90 days (4 [13%] versus 2 [6%]; odds ratio, 1.86 [0.37–9.50]; *P*=0.45) did not differ. All thromboembolic events occurred at least 2 weeks after study treatment, exclusively in participants not restarted on oral anticoagulation.

**CONCLUSIONS:**

In a smaller-than-intended NOAC-ICH patient sample, we found no evidence that TXA prevents hematoma expansion, but there were no major safety concerns. Larger trials on hemostatic treatments targeting an early treatment window are needed for NOAC-ICH.

**REGISTRATION:**

URL: https://clinicaltrials.gov; Unique identifier: NCT02866838.

Intracerebral hemorrhage (ICH) is a devastating disease with high mortality and morbidity.^1^ Oral anticoagulation is a well-defined complicating factor of ICH, resulting in increased risk of hematoma expansion (HE), mortality, and morbidity compared with patients with ICH without anticoagulation therapy.^2^ Novel, non–vitamin K antagonist oral anticoagulants (NOAC) are the mainstay of oral anticoagulation and have largely replaced vitamin K antagonists over the past decade. With the increasing use of NOAC, the proportion of NOAC-associated ICH is steadily increasing, too.^3^ Knowledge about NOAC-associated ICH is scarce, but retrospective observational studies reported high rates of HE (40%), high rates of in-hospital mortality (25%), and substantial disability.^4–6^

Currently, no evidence-based hemostatic treatment exists for NOAC-associated ICH.^7,8^ NOAC-specific reversal agents (idarucizumab for direct thrombin inhibitors and andexanet alfa for direct factor Xa inhibitors) have been approved by medical authorities but lack clear evidence from randomized controlled trials to be superior to any other standard of care.^9,10^ While guidelines recommend the use of NOAC-specific reversal agents and 4-factor prothrombin complex concentrate (4fPCC) to restore coagulation in NOAC-associated ICH, no robust evidence exists that these treatments limit HE or improve clinical outcomes.^7,8,11^

Tranexamic acid (TXA)—an antifibrinolytic drug—has been investigated in non–NOAC-associated ICH, most notably in the recent TICH-2 trial.^12^ Although its efficacy with regard to functional outcome has not been established, it may be modestly effective in reducing early death and limiting HE, particularly when administered early in patients at high risk for HE, and seems safe.^12–14^ With the advantages of wide availability, ease of administration, low cost, and distinct, non–NOAC-specific antifibrinolytic mechanism of action with potential for synergistic effects^15^ with the aforementioned procoagulant treatments currently recommended as standard of care, TXA seems an attractive candidate for the treatment of NOAC-associated ICH, for which it has not been tested so far.

Here, we report the results of TICH-NOAC (Treatment of Intracerebral Hemorrhage in Patients on Non–Vitamin K Antagonist Oral Anticoagulants With Tranexamic Acid), a randomized placebo-controlled trial investigating the effect of TXA on HE among patients with NOAC-associated ICH in addition to standard medical care.

## METHODS

### Data Availability

Trial data can be made available on reasonable request to the corresponding authors. Requests must be accompanied by a detailed study proposal, which must be approved by the corresponding authors and the trial steering committee, and checked for compatibility with regulatory requirements and patient informed consent.

### Study Design and Participants

TICH-NOAC was a double-blind, randomized, placebo-controlled, investigator-led phase 2 trial conducted at 6 stroke centers in Switzerland (Table S1). Adults with acute nontraumatic ICH were eligible for inclusion if they could be randomized within 12 hours of symptom onset (or, in patients with unknown symptom onset, if the time since last known to be well divided by 2 was <12 hours) and were taking any NOAC (apixaban ≥2.5 mg, dabigatran ≥110 mg, edoxaban ≥30 mg, or rivaroxaban ≥10 mg) with known last intake within 48 hours or proven persisting NOAC activity (ie, measurable NOAC plasma levels) using NOAC-specific coagulation assays. The 48-hour cutoff was chosen based on previous data demonstrating increased NOAC plasma levels up to 48 hours after the last intake among patients with NOAC-associated ICH.^16^ Exclusion criteria were severe preexisting disability (modified Rankin Scale [mRS] score >4); Glasgow Coma Scale (GCS) score <5; prior treatment with vitamin K antagonists; ICH known or suspected to be secondary to trauma, vascular malformation, tumor, or other underlying structural abnormality; pregnancy; planned neurosurgical hematoma evacuation within 24 hours; and pulmonary embolism or deep vein thrombosis within the preceding 2 weeks. Concurrent use of other hemostatic agents (eg, idarucizumab, andexanet alfa, and 4fPCC) was not an exclusion criterion.

TICH-NOAC was performed and reported in accordance with the study protocol and prespecified statistical analysis plan (under version control; latest version 1.4 [dated April 16, 2020] made available online at https://clinicaltrials.gov (NCT02866838) before database closure [March 22, 2022] and unmasking [March 24, 2022]; list of protocol changes is shown in the Supplemental Material), adhering to the guidelines of good clinical practice, the principles enunciated in the Declaration of Helsinki, and the Consolidated Standards of Reporting Trials (CONSORT) guideline for randomized controlled trials.^17^ Ethics approval was obtained for all Swiss sites from the lead Ethics Committee of Northwestern and Central Switzerland (EKNZ; 2016-01251) and the Swiss Agency for Therapeutic Products (SwissMedic; 2016DR3136) before study initiation. We obtained written informed consent from each participant if they were able to provide it. When participants were unable to consent themselves and no available information (eg, from patient decrees) indicated patients’ unwillingness to participate in clinical research studies, we obtained proxy consent from next of kin or legal representatives (as to the patients’ presumed will) or from independent physicians unrelated to the study (acting as patients’ custodians). In this case, we informed the participants about the trial and sought their consent as soon as their clinical status permitted.

### Randomization and Masking

Participants were randomly allocated to receive either TXA or matching placebo in a 1:1 ratio via a centralized, Web-based, secure procedure, with minimization for key prognostic factors (age, sex, National Institutes of Health Stroke Scale [NIHSS] score, and use of one of NOAC-specific reversal agents idarucizumab or andexanet alfa). To avoid predictable alternation of treatment allocation, and thus potential loss of allocation concealment, participants were allocated with a probability of 80% to the treatment group that would minimize between-group imbalances of the minimization factors. The randomization sequence was generated by the Clinical Trial Unit Basel. Participants, investigators involved in study assessments, and independent physicians involved in patient management were all masked to treatment allocation.

The pharmacy of the University Hospital Basel prepared, stored, and distributed (in numbered supplies) the investigational product to study sites in externally indistinguishable sealed treatment packs, each labeled with a unique number and containing either TXA or placebo (0.9% sodium chloride) in nonidentical commercial off-the-shelf ampoules as solution for intravenous infusion. Participants were assigned a treatment pack number via the randomization procedure. After randomization, the assigned sealed treatment pack was handed to an unmasked nurse not involved in study assessments, who unsealed it, added the ampoules into bags of normal saline, and administered the appropriately labeled masked infusion bags to the participant.

### Procedures

All participants were administered either intravenous TXA as an initial 1-g loading dose in 100-mL normal saline infused over 10 minutes and followed by another 1 g in 250-mL normal saline infused over 8 hours or placebo with an identical administration regimen. Participants received study treatment in addition to standard medical care, which included blood pressure management and use of other hemostatic agents at the discretion of the treating physicians according to local standards.

All clinical assessments for the trial were performed by local investigators. At randomization, investigators recorded the participants’ age, sex, and medical history (including information on NOAC treatment), as well as vital signs, NIHSS, GCS, and prestroke mRS. In-hospital follow-up was conducted at day 2, day 7, and on the day of death or hospital discharge, whichever occurred first, to collect information on adherence to study protocol (including whether all study treatment was administered and at which date and time) and the clinical course and management (including GCS, NIHSS, blood pressure management, neurosurgical interventions, medical treatments, care setting, and adverse events). All participants were followed-up with a final outpatient visit at 90±14 days to collect information on functional status using the mRS score, care setting, predefined adverse events (major thromboembolic events, seizures, and death), and medical treatments. When participants were unable to attend an in-person 90-day interview, a structured telephone interview with them, their next of kin, or caregivers was performed instead. All participants’ data were entered by local investigators into a secure, Web-based electronic case report form, maintained by the Clinical Trial Unit Basel. Independent on-site data monitoring was performed by the Clinical Trial Unit Basel according to a predetermined monitoring plan.

Baseline brain imaging was done by computed tomography (CT) as part of routine care before randomization. A follow-up CT scan was done after 24±3 hours to assess HE. All imaging assessments of deidentified scans were performed by an independent central reader (G.M.K.), masked to treatment allocation and clinical outcomes of the participants, and entered into the electronic case report. Imaging assessment included characterization of the intracerebral hematoma on baseline and follow-up nonenhanced thin-sliced (1 to 1.5 mm) CT scans (hematoma volume, location, intraventricular extension) and assessment of spot sign^18^ (ie, active contrast extravasation on baseline CT angiography, when available). For hematoma volumetry, planimetric measurements were calculated based on hand-drawn polygonal regions of interest encompassing the hyperdense hematoma in each slice using the Syngo.via software (Siemens Healthcare, Germany). Independent validation of the volumetric assessments was performed by a second masked rater (B.M.S.) using different software (3D Slicer^19^) with a threshold-based (44–100 Hounsfield units) semiautomated segmentation approach with manual correction, as in prior research.^20^ In case of disagreement about the presence versus absence of HE (ie, the primary outcome), a third tie-breaking rater (D.J.S.) was involved.

### Outcomes

The primary outcome was the presence of HE on follow-up imaging at 24 (±3) hours, defined as intracerebral hematoma volume increase by at least 33% or 6 mL from baseline, in accordance with prior research.^12,14^

Prespecified secondary outcomes were symptomatic HE, defined as HE with neurological deterioration (worsening of NIHSS score by at least 4 points or GCS score by at least 2 points) or death within 7 days; absolute hematoma volume change by 24±3 hours; ordinal mRS score, mRS score 0 to 4, and mRS score 0 to 3 at 90 days; in-hospital death; death within 90 days; major thromboembolic events (ischemic stroke, myocardial infarction, or deep vein thrombosis/pulmonary embolism defined as clinical syndromes with supporting paraclinical evidence) within 90 days; and neurosurgical intervention up to day 2.

Prespecified subgroups were onset-to-treatment time (≤6 versus >6 hours), baseline hematoma volume (≤30 versus >30 mL), hematoma location (originally categorized into deep, lobar, and cerebellar but modified to a dichotomous analysis of nonlobar versus lobar due to small number of participants with cerebellar hemorrhage), intraventricular hemorrhage (absent versus present), GCS (≤12 versus >12), age (originally with a cutoff of 70 years but modified to ≤80 versus >80 years due to small number of participants aged ≤70 years), and spot sign (absent versus present).

### Statistical Analysis

The sample size for TICH-NOAC was calculated at 109 participants to be able to detect a significant relative difference of 50% in the rate of HE (27% in the treatment versus 54% in the control arm) with a 5% dropout rate and 80% power at a 2-sided α of 5%. The control group HE rate was based on data from vitamin K antagonist–associated ICH,^21,22^ as virtually no data existed for NOAC-associated ICH at the time of protocol development. In the absence of previous data on the effect of TXA on HE, we based our sample size calculation on feasibility of recruitment and chose a 50% relative reduction in the rate of HE as the targeted effect size based on data from the only trial of hemostatic treatment that was available at the time of protocol development, which had investigated 4fPCC versus fresh frozen plasma in vitamin K antagonist–associated ICH and showed a 50% relative reduction in HE rate with 4fPCC.^23^ Justified by lack of previous efficacy data, our protocol allowed for an adaptive sample size increase up to 218 participants depending on the results of an interim analysis after reaching 82 participants. Due to lack of funding, TICH-NOAC was terminated early before reaching this.

We evaluated the interrater reliability of the primary outcome assessment using the κ statistic (with a value of 0 indicating chance agreement and 1 perfect agreement, while values of 0.61–0.80 and over 0.80 indicate substantial and near perfect agreement, respectively). Following the prespecified statistical plan and in accordance with prior research,^14^ we compared the primary outcome between treatment and control arms using binary logistic regression adjusted for baseline hematoma volume (as a continuous variable) for the primary analysis. In a post hoc sensitivity analysis, we additionally adjusted the model for time from onset to baseline imaging. We imputed missing primary outcome data assuming worst possible outcome, as in prior research.^14^

In secondary analyses, we compared the secondary binary outcomes symptomatic HE, dichotomized mRS, and death between the treatment groups using binary logistic regression adjusted for baseline hematoma volume. The remaining binary secondary outcomes were compared using unadjusted Firth regression as appropriate, depending on the small number of outcome events. We additionally analyzed 90-day mRS over its entire ordinal range using shift analysis adjusted for baseline hematoma volume, assessing the validity of the proportional odds assumption with visual inspection of the mRS distribution and using both the approximate likelihood ratio and Brant test. We compared the absolute hematoma volume change between treatment groups using median regression adjusted for baseline hematoma volume with bootstrapped SE estimation and 1000 repetitions, since the assumptions of linear regression were violated even after log-transformation. Further not prespecified exploratory analyses of additional binary outcomes were done using logistic regression adjusted for baseline hematoma volume.

We analyzed the primary outcome in prespecified subgroups using binary logistic regression adjusted for baseline hematoma volume and appropriate interaction terms. We also explored the following not prespecified subgroups: systolic blood pressure on admission (≤170 versus >170 mm Hg), concurrent use of 4fPCC (used versus not used), and ANNEXa-I (ongoing randomized controlled trial investigating andexanet alfa in patients with factor Xa inhibitor–associated ICH; https://clinicaltrials.gov; NCT03661528) eligibility status (eligible versus ineligible). Methodological details on the latter subgroup are given in the Supplemental Material. In an additional not prespecified exploratory analysis, we investigated the interaction of study treatment with onset-to-treatment time, both as a continuous measure and dichotomized using different time cutoffs (3, 4.5, and 7.5 hours, in addition to the prespecified 6-hour cutoff). Finally, we did the following not prespecified exploratory analyses post hoc: we investigated the interaction of study treatment with admission NOAC plasma level and time from last NOAC intake to study treatment (both as continuous variables) and explored center effects by assessing for treatment-by-center interaction (top-recruiting center Basel versus all other centers grouped together). All subgroup analyses were regarded as hypothesis generating only.

The nominal level of significance for all analyses was a *P* value <0.05. No adjustment was made for multiple testing. Analyses were done on an intention-to-treat basis as prespecified in the statistical plan and were repeated in the per-protocol population (after exclusion of participants who did not receive treatment as per protocol). A.A.P. and S.S. did the statistical analyses using Stata, version 17.0 (StataCorp LLC, College Station, TX).

The trial was overseen by a Steering Committee and run by an Executive Committee based at the University Hospital Basel and chaired by P.A.L. An independent data safety monitoring board was planned but was not appointed due to early trial termination. The trial was prospectively registered at https://clinicaltrials.gov (August 15, 2016; NCT02866838) before enrolling the first patient.

## RESULTS

**Figure 1.**
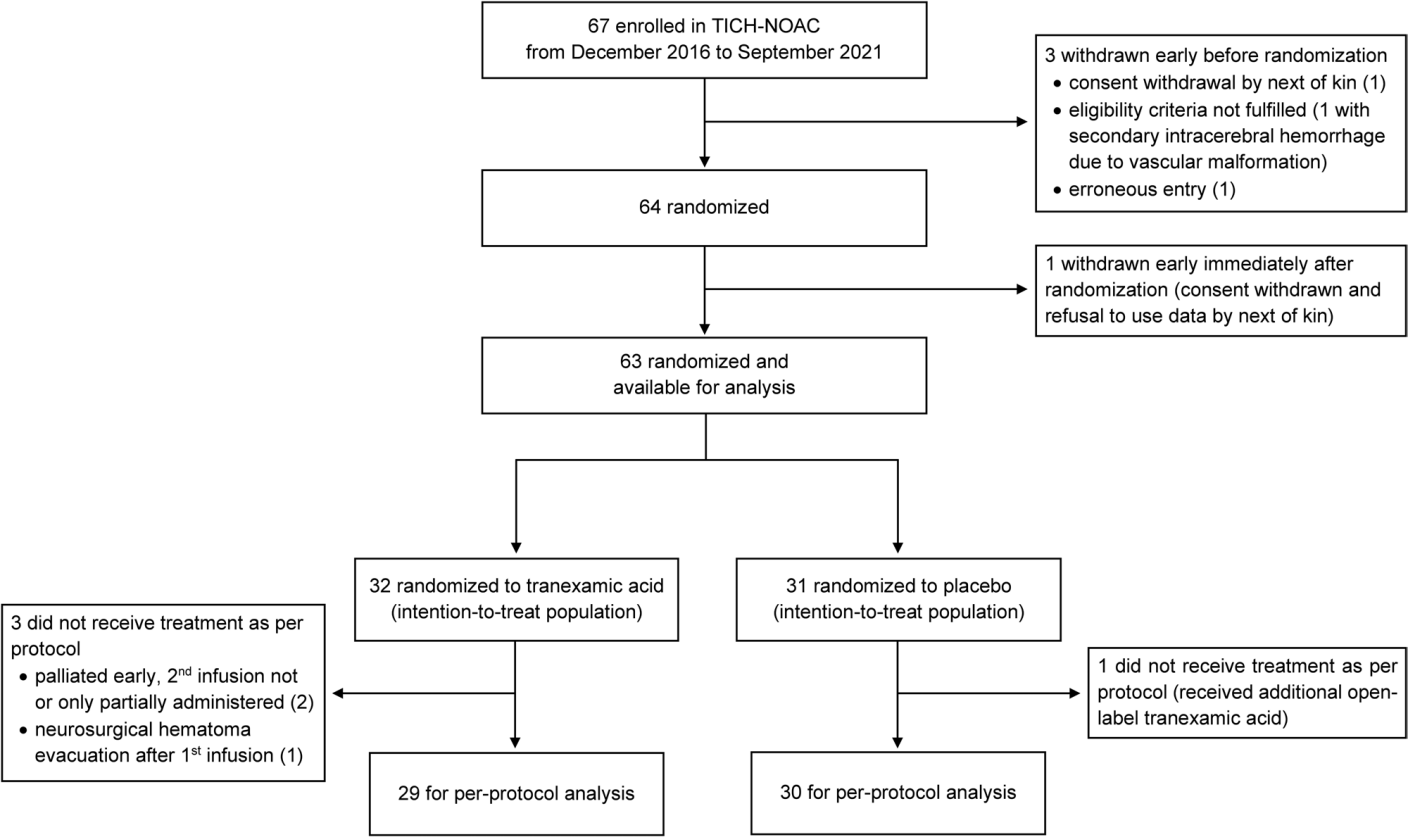
**Trial profile.** Comprehensive screening data for all consecutive patients with non–vitamin K antagonist oral anticoagulant-associated intracerebral hemorrhage were not available because study staff availability for screening varied across recruiting centers and across time. TICH-NOAC indicates Treatment of Intracerebral Hemorrhage in Patients on Non–Vitamin K Antagonist Oral Anticoagulants With Tranexamic Acid.

Between December 12, 2016, and September 30, 2021, 67 patients were enrolled in TICH-NOAC. Of those, 3 were withdrawn early before randomization and 1 was withdrawn immediately after randomization, without receiving study treatment and with their next of kin refusing the use of their data for analysis. This amounted to 63 evaluable randomized participants, 32 assigned to TXA and 31 to placebo. All but 4 received treatment as per protocol (Figure 1). All participants had follow-up imaging, but 2 who died before follow-up imaging could be acquired (1 TXA and 1 placebo) and were assigned the HE outcome as prespecified in the statistical plan. Follow-up imaging was done at a median (interquartile range) of 24 (21.6–24.8) hours after baseline imaging (25.9 [23.6–30.6] hours after symptom onset). All participants had complete 90-day follow-up, and allocation masking was not broken in any participant. Figure S1 shows the recruitment of evaluable randomized participants stratified by recruiting site over time.

The median age of participants was 82 years (interquartile range, 76–86), and 25 (40%) were women. All were taking direct factor Xa inhibitors (apixaban, edoxaban, or rivaroxaban), and none was taking direct thrombin inhibitors (dabigatran). The most common NOAC was rivaroxaban (49 participants; 78%), and atrial fibrillation was the leading indication for anticoagulation therapy (55; 87%). The median NIHSS score was 13 (6–19), median hematoma volume was 11.5 mL (4.8–27.4), and hematomas were more commonly nonlobar (44; 70%). The median time from onset to study treatment was 5 hours (2.8–9.1). No participants received NOAC-specific reversal agents (neither idarucizumab nor andexanet alfa), while 41 (65%) received concomitant treatment with 4fPCC. Participant characteristics were reasonably balanced between the groups, except for lobar ICH being more common and time to study treatment being longer in the TXA group (Table 1).

**Table 1.**
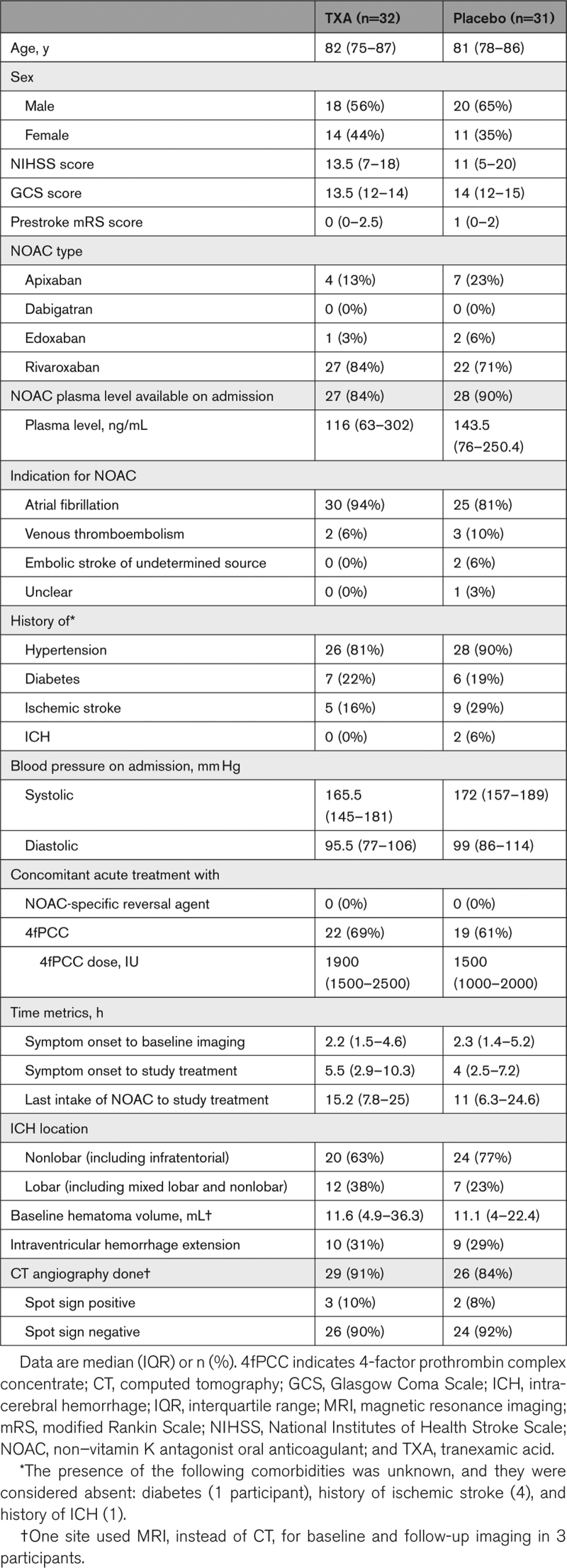
Baseline Characteristics of the Intention-to-Treat Population

Interrater reliability of the primary outcome assessment (ie, the presence or absence of HE on the follow-up scan) between 2 raters was almost perfect (90.5% agreement; κ, 0.81), and disagreement in 6 participants was resolved by the third tie-breaking rater. In total, 26 participants (41%) had HE. The primary outcome did not differ between the treatment arms, with 12 of 32 participants in the TXA (38%) and 14 of 31 in the placebo arm (45%) showing HE (odds ratio [OR] adjusted for baseline hematoma volume, 0.63 [95% CI, 0.22–1.82]; *P*=0.40; unadjusted OR, 0.73 [0.27–1.99]; *P*=0.54; post hoc sensitivity analysis adjusting for both baseline hematoma volume and time from onset to baseline imaging: OR, 0.64 [0.22–1.89]; *P*=0.42).

**Figure 2.**
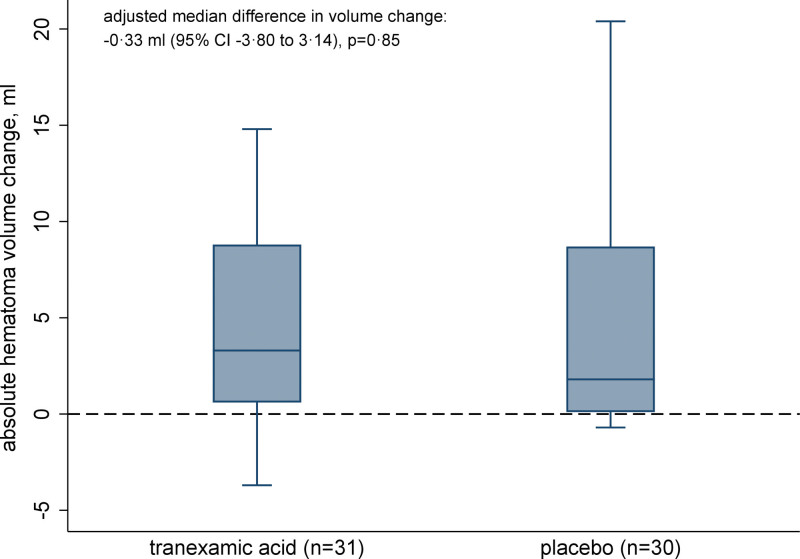
**Box plot of absolute hematoma volume change from baseline to follow-up imaging in the intention-to-treat population.** Boxes show median and interquartile range (IQR), the whiskers show the full range of values excluding outliers over 1.5× the IQR beyond the IQR limits. Not included are 2 participants without follow-up imaging (1 assigned to tranexamic and 1 to placebo). A histogram of the absolute hematoma volume change is given in Figure S3.

None of the prespecified secondary outcomes differed between the treatment arms (Table 2; Figure 2; Figures S2 and S3). We observed no difference in major thromboembolic events in participants with allocation to TXA and concomitant treatment with 4fPCC. All major thromboembolic events occurred no earlier than 2 weeks after ICH onset exclusively in patients who had not been restarted on oral anticoagulation at the time of the thromboembolic event (Table S2). In exploratory analyses of additional nonprespecified outcomes, there was no difference between the arms in the rate of participants with seizures within 90 days (6 participants in the TXA [19%] and 4 in the placebo arm [13%]; adjusted OR, 1.62 [0.41–6.50]; *P*=0.49) nor in the rate of participants with any serious adverse events (including major thromboembolic events, seizures, and death collected up to 90 days and any serious adverse events collected up to day 7; 24 participants in the TXA [75%] and 20 participants in the placebo arm [65%]; adjusted OR, 1.43 [0.45–4.47]; *P*=0.54).

**Table 2.**
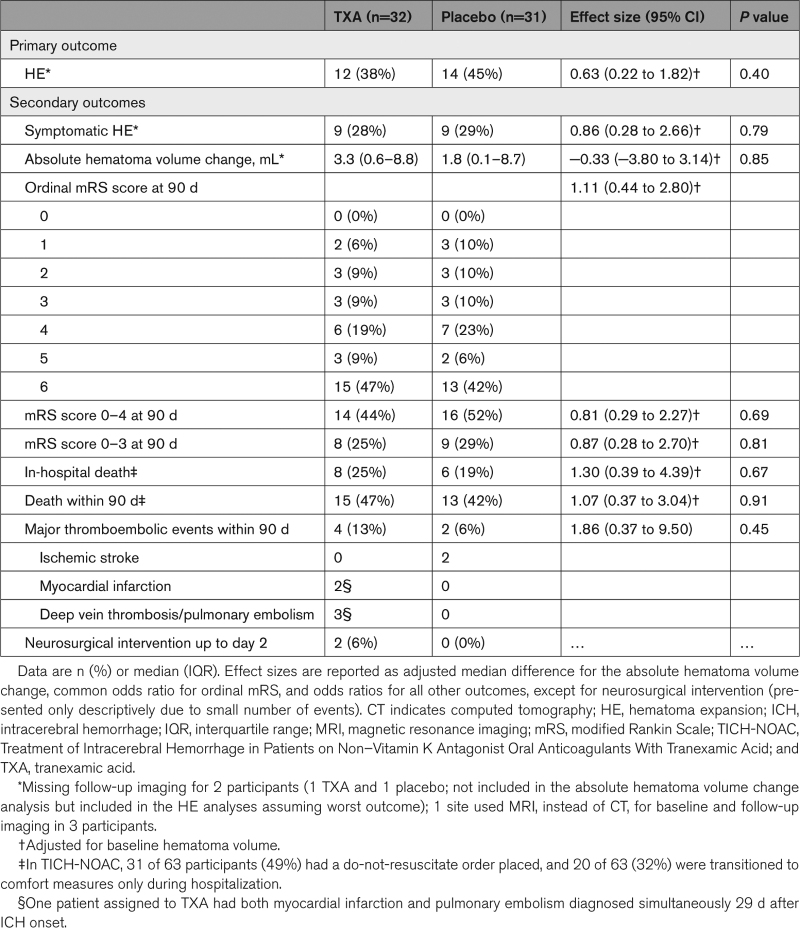
Primary and Secondary Outcomes in the Intention-to-Treat Population

**Figure 3.**
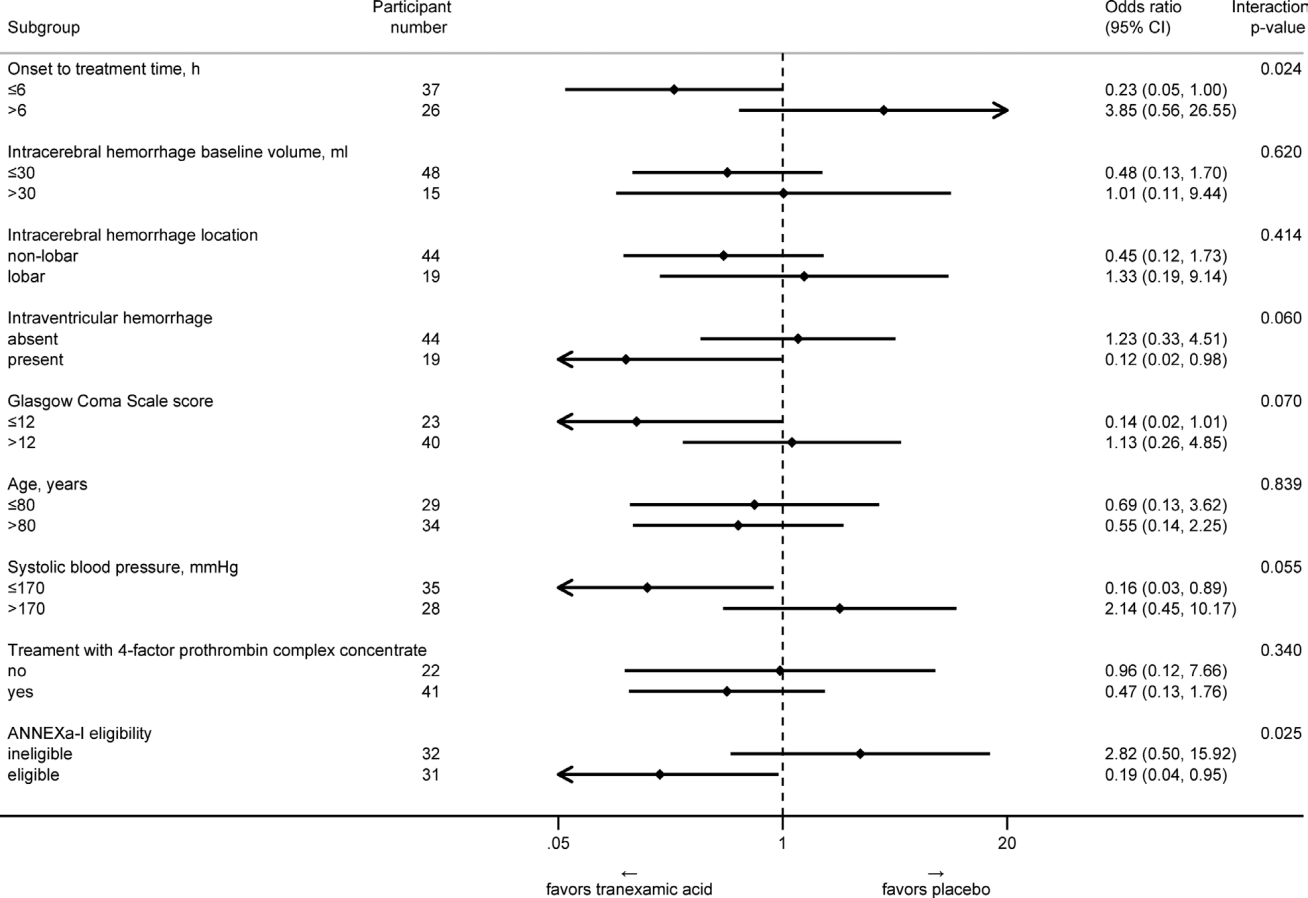
**Primary outcome by subgroups in the intention-to-treat population.** All subgroups were predefined except for systolic blood pressure, concomitant treatment with 4-factor prothrombin complex concentrate, and ANNEXa-I (Andexanet Alfa in Acute Intracranial Hemorrhage in Patients Receiving an Oral Factor Xa Inhibitor) eligibility. All odds ratio estimates are adjusted for baseline hematoma volume and were derived in each subgroup analysis from 2 separate models (1 for each category of the binary subgroup, fitted after excluding participants of the other category).

Subgroup analyses yielded imprecise estimates with wide CIs due to small participant numbers. Of the investigated subgroups, there was a signal for interaction of study treatment with onset-to-treatment time (dichotomized at the prespecified 6-hour cutoff; *P*_interaction_=0.024) and ANNEXa-I eligibility (*P*_interaction_=0.025), suggesting a potential effect of TXA on limiting HE in participants with onset-to-treatment time ≤6 hours and those fulfilling the ANNEXa-I eligibility criteria. The analyses of interaction with the presence of intraventricular hemorrhage, GCS score, and admission systolic blood pressure tended to favor TXA in participants with intraventricular hemorrhage extension, GCS ≤12, and systolic blood pressure ≤170 mm Hg. There was no signal for interaction with concomitant 4fPCC treatment or any of the remaining subgroups (Figure 3). Due to the small participant number in the subgroups, no analysis on spot sign (positive in 5 of 55 participants with available CT angiography) was possible. The exploratory analysis of interaction with onset-to-treatment time as a continuous measure had a *P* value of 0.036, suggesting a potential interaction with study treatment on its effect on the primary outcome, with earlier treatment favoring TXA. Interaction analyses of onset-to-treatment time dichotomized at different time cutoffs yielded qualitatively consistent results with the prespecified analysis at the 6-hour cutoff (Figure S4). There was no evidence for interaction of study treatment with admission NOAC plasma level (available in 55 participants; *P*_interaction_=0.351) or time from last NOAC intake to study treatment (*P*_interaction_=0.227) on their effect on the primary outcome. Finally, there was no clear signal for treatment-by-center interaction (*P*_interaction_=0.081).

Baseline characteristics, outcomes, and subgroup analyses in the per-protocol population were consistent with those of the intention-to-treat population (Tables S3 and S4; Figures S5 through S7).

## DISCUSSION

In this first randomized controlled trial of TXA versus placebo in patients with NOAC-associated ICH, we found no evidence that TXA limits HE nor that it improves clinical outcomes by 90 days. However, there were no major safety concerns for the use of TXA, and treatment might hold potential for preventing HE in selected patient subgroups, particularly those treated early after ICH onset.

The hemostatic effect reflects the balance between the opposing processes of thrombin generation through the coagulation cascade leading to fibrin clot formation and endogenous fibrinolysis leading to fibrin clot disintegration. TXA does not affect the coagulation factors thrombin or factor Xa, which are the targets of the NOAC, but blocks endogenous fibrinolysis, thus delaying fibrin disintegration and accelerating and stabilizing fibrin clot formation,^24^ a mechanism of action that is not specific to NOAC-associated bleeding. TXA was recently tested for acute ICH treatment in several randomized controlled trials,^12,14,25^ with further trials ongoing.^26^ Meta-analyses of the so far available evidence, driven mostly by the large TICH-2 trial,^12^ showed that TXA may be modestly effective in limiting HE, particularly when administered early in patients at high risk for HE.^13^ However, these trials excluded patients with NOAC-associated ICH, who are becoming increasingly more common in clinical practice.^3^

Currently, no randomized evidence exists for the hemostatic treatment of NOAC-associated ICH. While recent guidelines^7,8^ make weak recommendations for the use of NOAC-specific reversal agents (ie, idarucizumab^9^ for dabigatran-associated ICH and andexanet alfa^10^ for factor Xa inhibitor–associated ICH) and 4fPCC based mostly on hemostasiological data regarding efficacy in anticoagulation reversal and uncontrolled observational data on clinical outcomes, whether these treatments limit HE or improve clinical outcomes compared with placebo remains unproven.^11^ Considering the antifibrinolytic mechanism of action of TXA, which is distinct from the procoagulant effect of the aforementioned hemostatic treatments, and the few available experimental data on the combination of these hemostatic strategies showing potential synergistic hemostasiological effects,^15^ we designed TICH-NOAC to test TXA in addition to standard medical care, which could include other hemostatic treatments according to local standards and at the discretion of the treating physicians. Reflecting clinical practice at the time of the study, approximately two-thirds of TICH-NOAC participants received additional treatment with 4fPCC. No TICH-NOAC participants received NOAC-specific reversal agents, as andexanet alfa was not available in Switzerland during the study period and no patient on dabigatran was enrolled in our trial.

Although TICH-NOAC did not reach its target sample size, which precludes any definitive conclusions, there was no clear signal for potential efficacy in any of the predefined outcomes. The rate of HE, which we chose as our primary dichotomous outcome based on considerations discussed elsewhere,^14^ was high in both treatment groups, and only 7%—nonsignificantly—lower with TXA compared with placebo. The rate of death by 90 days was also quite similar in both treatment arms at over 40%. This high rate of poor outcomes is consistent with previous observational data on NOAC-associated ICH^4–6^ and underlines the pressing unmet need for effective treatments. Of note, our subgroup analysis showed no signal for a synergistic effect between TXA and 4fPCC, which might have been expected considering their distinct hemostatic mechanisms (the former blocking fibrinolysis and the latter counteracting the anticoagulant effect of the NOAC by replenishing coagulation factors). Whether NOAC-specific hemostatic treatments might be more effective than nonspecific ones, such as TXA or even its combination with 4fPCC, remains to be determined. The ongoing ANNEXa-I trial will provide evidence about the efficacy of specific reversal of factor Xa inhibitors with andexanet alfa in NOAC-associated ICH. In the meantime, our finding of a potential beneficial effect of TXA in the subgroup of participants who would have qualified for ANNEXa-I strengthens confidence in the appropriateness of this trial’s selection criteria.

Although our subgroup analyses, limited by the small participant numbers leading to high imprecision, are highly exploratory, their hypothesis-generating results might help inform the design of future trials. Notably, we found a signal for interaction of treatment with time from onset, suggesting that there might be potential for benefit from TXA with earlier treatment administration. This also seems to drive the aforementioned association in the subgroup of ANNEXa-I–eligible participants and adds to the accumulating evidence from recent trials on TXA in spontaneous non–NOAC-associated ICH but also traumatic brain injury and other severe hemorrhage, that early treatment is crucial to achieve better outcomes.^13,27,28^ It is, therefore, possible that the absence of a clear signal for efficacy in our trial, which included participants up to 12 hours from ICH onset and had an actual median time to study treatment of 5 hours, may be partly attributed to dilution of a potential treatment effect by inclusion of patients later after ICH onset, for whom benefit may no longer be attainable. Indeed, HE is known to occur mostly in the first few hours after ICH onset^29^ and might have been missed in TICH-NOAC participants treated later. Accordingly, ongoing and planned trials of TXA in non–NOAC-associated ICH are targeting an earlier time window (STOP-MSU: https://clinicaltrials.gov; NCT03385928; TICH-3: https://www.isrctn.com/; ISRCTN97695350), as does the ANNEXa-I trial of andexanet alfa in NOAC-associated ICH.

The analysis of interaction with admission systolic blood pressure showed a weak signal that participants with lower systolic blood pressure (≤170 mm Hg) might be more responsive to TXA. This is consistent with the findings of the TICH-2 trial, which reported a similar interaction of treatment with blood pressure.^12^ Given the known association between high admission systolic blood pressure and increased risk of HE,^30^ a potential explanation for these findings may be that HE might have already occurred in participants with high blood pressure by the time of treatment, leaving those with lower blood pressure to benefit more from treatment. Finally, our subgroup analyses showed a weak signal in favor of TXA among participants with intraventricular hemorrhage extension and lower GCS score (≤12). Why TXA would perform better in these apparently more severely affected patients^31^ is unclear. Further research is needed to confirm and explore the basis for these signals for potential interactions, which are by no means conclusive and should be interpreted with caution.

Importantly, our study showed no signal for a major safety concern for the use of TXA in addition to standard care in patients with NOAC-associated ICH, consistent with previous evidence for the safety of TXA from trials on non–NOAC-associated ICH.^12,13,32^ Compared with these trials, where major thromboembolic events within 90 days occurred at a rate of about 3%,^13^ the much higher rate observed in TICH-NOAC of about 10% is consistent with prior reports^9,10^ and is probably attributable to the differences of the populations studied: a priori, patients taking NOAC are at high risk for thromboembolic events, which is accentuated after the abrupt discontinuation of NOAC treatment due to the acute ICH. All major thromboembolic events were observed in participants who had not been restarted on oral anticoagulation and occurred at least 2 weeks after ICH onset, which makes them unlikely to be related to study treatment. There were no excess thromboembolic events in participants treated with both TXA and 4fPCC. Notably, additional nonprespecified analyses revealed no differences in the rate of participants with seizures or any serious adverse events between the treatment arms, consistent with the known safety profile of TXA in non–NOAC-associated ICH.^12^ However, the numerically—albeit nonsignificantly—higher rate of adverse events in the TXA arm merits further investigation in future larger trials, considering the small sample size of TICH-NOAC.

The strengths of TICH-NOAC are as follows: (1) this is the first randomized trial reporting on the hemostatic treatment of NOAC-associated ICH, (2) robust methodology including a double-blind placebo-controlled design, and (3) robust assessment of the primary outcome by 3 raters, which limit the risk of bias. The most important limitations of TICH-NOAC are as follows: (1) failure to reach its target sample size and (2) underpowered sample size. A number of factors led to the premature discontinuation of TICH-NOAC, including lack of additional funding and—most notably—the negative impact of the COVID-19 pandemic on research activities,^33^ which slowed participant recruitment in active sites and precluded the originally planned expansion of our trial to international sites. Although the neutral results of TICH-NOAC should be interpreted cautiously in light of our small sample size, in retrospect, it seems unlikely that our trial could have met the primary end point, considering that (1) the expected HE rate of 54% in the placebo arm was apparently overestimated, with newer data made available after protocol development indicating an HE rate of about 40% in NOAC-associated ICH,^4,5^ and (2) the effect of TXA was probably overestimated at the hypothesized relative HE reduction of 50%, with newer trial data on TXA in non–NOAC-associated ICH indicating a much more modest effect on HE (<15% relative reduction).^12,14,25^ Although our finding of a 7% absolute reduction in the HE rate is in line with these reports, for TICH-NOAC to have been able to demonstrate such a smaller effect with confidence, far more participants in the order of a few thousand would have been required (Figure S8). Even so, whether such a small effect on HE would translate to better clinical outcomes remains unclear.^34^ Furthermore, although our protocol allowed for the concurrent use of other hemostatic treatments based on considerations laid out above, we cannot exclude that this choice may have further attenuated our trial’s ability to detect any effect of TXA on HE. Baseline imbalances in the prevalence of lobar ICH and timing of treatment may have additionally diluted any treatment effect of TXA. Finally, our subgroup analyses—being underpowered and imprecise—should only be considered highly exploratory and interpreted with caution.

In summary, our study found no evidence that TXA prevents HE in patients with NOAC-associated ICH, although there were no major safety concerns with TXA and treatment might hold potential for benefit in selected patient subgroups, particularly those treated early after ICH onset. Further trials on hemostatic treatments are urgently needed to improve outcomes in NOAC-associated ICH and should probably target an early treatment window. Including the increasingly important population of patients taking NOAC in ongoing or planned large-scale trials of TXA in ICH is warranted.

## ARTICLE INFORMATION

### Acknowledgments

We are grateful to the Clinical Trial Unit Basel, Department of Clinical Research of the University of Basel, and the pharmacy of the University Hospital Basel for their support in the conduct of TICH-NOAC (Treatment of Intracerebral Hemorrhage in Patients on Non–Vitamin K Antagonist Oral Anticoagulants With Tranexamic Acid; Nicole Bruni for programming and data management; Klaus Ehrlich for data monitoring; Bettina Bannert for trial registration; Stefanie Deuster for preparing study treatment packs). We thank all TICH-NOAC investigators, listed in Table S1, for their contribution to patient recruitment.

### Sources of Funding

TICH-NOAC (Treatment of Intracerebral Hemorrhage in Patients on Non–Vitamin K Antagonist Oral Anticoagulants With Tranexamic Acid) was funded by the Swiss National Science Foundation (grant number 32003B_163378), the Stroke Fund, Neurology Fund, and Scientific Fund of the University Hospital Basel. The funders of the study had no role in study design, data collection, data analysis, data interpretation, or writing of the report.

### Disclosures

Dr Polymeris: funding from Swiss Academy of Medical Sciences (SAMS)/Bangerter-Rhyner-Foundation, Swiss Heart Foundation (SHF). Dr Siepen: funding from SAMS/Bangerter-Rhyner-Foundation. Dr Tsakiris: unrestricted research grants and lecture honoraria from Bayer Schweiz AG, Pfizer, Daiichi Sankyo, Takeda. Dr Sprigg: grants from the National Institute of Health Research. Dr Thilemann: travel grants from Bristol Myers Squibb (BMS)/Pfizer. Dr Wagner: funding by the Doc.Mobility instrument of the Swiss National Science Foundation (SNSF), Prof Dr med Karl und Rena Theiler-Haag Stiftung. Dr Traenka: research grants from SHF; personal research scholarships from the Novartis Foundation for biological and medical research, Freiwillige Akademische Gesellschaft Basel, Bangerter-Rhyner Foundation, and University of Basel; travel grants from Bayer. Dr Gensicke: research support from SNSF; advisory board honoraria from Daiichi Sankyo; funding for travel from BMS/Pfizer, and AbbVie. Dr De Marchis: support from SNSF (Nr.32003B_200573 and Nr.PBBEP3_139388); Spezialprogramm Nachwuchsförderung Klinische Forschung, University of Basel; Science Funds (Wissenschaftspool) of the University Hospital Basel; SHF; ProPatient Foundation Basel; Bangerter-Rhyner-Stiftung; Swisslife Jubiläumsstiftung for Medical Research; Swiss Neurological Society; Fondazione Dr. Ettore Balli; DeQuervain research grant; Thermo Fisher GmbH; Novartis grant; travel honoraria from Bayer, BMS/Pfizer; speaker honoraria from Bayer, Medtronic; consultant honoraria from Bayer, Novartis; member of the Steering Committee of PACIFIC Stroke (https://clinicaltrials.gov; NCT04304508); Industry payments made to the research fund of the University Hospital Basel. Dr Bonati: compensation from BMS for consultant services; compensation from Amgen for consultant services; grants from SHF; grants from Stiftung zur Förderung der gastroenterologischen und allgemeinen klinischen Forschung sowie der medizinischen Bildauswertung; travel support from Bayer; grants from University of Basel; grants from SNSF; grants from AstraZeneca; compensation from AstraZeneca for consultant services; travel support from AstraZeneca; compensation from Bayer for consultant services; and compensation from Claret Medical, Inc, for data and safety monitoring services. Dr Fischer: financial support for the SWIFT DIRECT trial from Medtronic; research grants from Medtronic BEYOND SWIFT registry, SNSF, SHF; compensation from Biogen and Boehringer Ingelheim for expert witness services and consulting fees from Medtronic, Stryker, CSL Behring (fees paid to the institution); membership of a Data Safety Monitoring Board for the IN EXTREMIS trial, TITAN trial; Portola (Alexion) Advisory board (fees paid to the institution); compensation from Phenox, Inc, for end point review committee services; grants from Phenox, Inc, Rapid Medical, Penumbra; Vice Presidency of the Swiss Neurological Society. Dr Wegener: research funds by SNSF, UZH Clinical Research Priority Program stroke, Zurich Neuroscience Center, Baugarten Foundation; speaker honoraria from Amgen, Springer, Teva Pharma; consultancy fee from Bayer, Novartis. Dr Baumgartner: research funds from SAMS/Bangerter-Rhyner-Foundation; funding for travel and conference fees from BMS/Pfizer. Dr Engelter: funding for travel or speaker honoraria from Bayer, Boehringer Ingelheim, Daiichi Sankyo; scientific advisory boards for Bayer, Boehringer Ingelheim, BMS/Pfizer, MindMaze; editorial board of Stroke; research funding to his institutions from Pfizer (educational grant), Stago (compensation for educational efforts), Daiichi Sankyo, Science Funds (Wissenschaftsfonds) University Hospital Basel, University of Basel, Wissenschaftsfonds Rehabilitation University Department for Geriatric Medicine Felix Platter, Freiwillige Akademische Gesellschaft Basel, SHF, SNSF. Dr Seiffge: research support from Science Funds and Stroke Funds University Hospital Basel, SNSF, Swiss Society of Neurology, Bangerter-Rhyner-Foundation, Daiichi Sankyo, Stago (compensation for educational efforts); thrombosis research prize from Bayer Foundation; advisory boards and consultancy for Portola/Alexion/AstraZeneca, Bayer, VarmX. Dr Peters: research support from SHF, SNSF; speaker honoraria from Vifor Pharma, OM Pharma; scientific advisory boards for Daiichi Sankyo, AstraZeneca, OM Pharma; compensation from Medtronic, Novo Nordisk, Vifor Pharma for expert witness services; compensation from Boehringer Ingelheim, AstraZeneca, Novo Nordisk for other services. Dr Lyrer: research support from SNSF, SHF, Research Funds Neurology University Hospital Basel; advisory boards for Boehringer Ingelheim, Bayer, Recordati SA, Daiichi Sankyo; travel support from Pfizer; compensation from Biogen for other services; compensation from Pfizer, Boehringer Ingelheim for consultant services. The other authors report no conflicts.

### Supplemental Material

Supplemental Methods

Tables S1–S4

Figures S1–S8

## Supplementary Material
