# Supplementary material for: Tranexamic Acid for Intracerebral Hemorrhage in Patients on Non-Vitamin K Antagonist Oral Anticoagulants (TICH-NOAC): A Multicenter, Randomized, Placebo-Controlled, Phase 2 Trial
Source: Stroke. 2023 Jul 19;54(9):2223–34. doi: 10.1161/STROKEAHA.123.042866 (PMC10453353; doi:10.1161/STROKEAHA.123.042866)
Supplement: Supplementary file 1 [file str-54-2223-s001.pdf]

Tranexamic acid for IntraCerebral Hemorrhage in patients on Non-vitamin K antagonist Oral AntiCoagulants (TICH-NOAC): a multicenter, randomized, placebo-controlled, phase 2 trial

Supplementary Material

Supplementary Methods 1

Supplementary Table S1. Participating sites and investigators in alphabetical order 2

Supplementary Table S2. Major thromboembolic events and re-initiation of oral anticoagulation in the intention-to-treat population 3

Supplementary Table S3. Baseline characteristics of the per-protocol population 4

Supplementary Table S4. Primary and secondary outcomes in the per-protocol population 5

Supplementary Figure S1. Recruitment of evaluable randomized participants stratified by recruiting site over time 6

Supplementary Figure S2. Shift plot of modified Rankin Scale score at 90 days in the intention-to-treat population 7

Supplementary Figure S3. Histogram of the absolute hematoma volume change from baseline to follow-up imaging in the intention to treat population 8

Supplementary Figure S4. Primary outcome by subgroups according to onset-to-treatment time in the intention-to-treat population (exploratory analysis) 9

Supplementary Figure S5. Boxplot of absolute hematoma volume change from baseline to follow-up imaging in the per-protocol population 10

Supplementary Figure S6. Shift plot of modified Rankin Scale score at 90 days in the per-protocol population 11

Supplementary Figure S7. Primary outcome by subgroups in the per-protocol population 12

Supplementary Figure S8. Sample size calculation assuming the observed effect 13

Supplementary Methods

List of protocol changes:

- Specification of consent procedure for patients unable to consent themselves (version 1·1, dated September 2, 2016; details given in Methods)
- Clarification that standard medical care may include the use of NOAC-specific reversal agents (version 1·1, dated September 2, 2016; version 1·2, dated November 28, 2016)
- Clarification that proven persisting NOAC activity is defined as measurable NOAC plasma level (version 1·2, dated November 28, 2016)
- Change regarding imaging modality requirements from "In centers where MRI is used as standard first-line imaging for stroke patients on admission, no additional CT scan is needed. In this case, the follow-up imaging on day 2 will be also MRI." to "If a patient received MRI scan on admission as first neuroimaging, the patient will undergo a baseline CT scan after inclusion in the trial […]." (version 1·3, dated May 12, 2017)
- Change in participating sites list: addition of Cantonal Hospital Lucerne and omission of Geneva, Lausanne, and Lugano (version 1·3, dated May 12, 2017); addition of Klinik Hirslanden Zurich (version 1·4, dated April 16, 2020)
- Change of the role of Prof. Philippe Lyrer from "Co-Chief Investigator" to "Sponsor-Representative and Coordinating Investigator"; Prof. Stefan Engelter from "Co-Chief Investigator" to "Deputy Coordinating Investigator and Local Principal Investigator University Hospital Basel"; Prof. Nils Peters from "Executive Trial Manager" to "Deputy Coordinating Investigator and Executive Trial Manager; Local Principal Investigator Klinik Hirslanden Zurich" (version 1·4, dated April 16, 2020)
- Due to changes in institutional licensing policies, different software (Syngo.via; 3D Slicer) was finally used for hematoma volumetry as the one originally planned in the protocol (Aquarius, TeraRecon).

Details on additional non-prespecified exploratory subgroups:

To assess for modification of the study treatment effect according to participants' eligibility for the ongoing ANNEXa-I trial ("A Randomized Clinical Trial of Andexanet Alfa in Acute Intracranial Hemorrhage in Patients Receiving an Oral Factor Xa Inhibitor"), we categorized study participants to ANNEXa-I eligible vs ineligible according this trial's eligibility criteria, available online at https://clinicaltrials.gov/ct2/show/NCT03661528. Key ANNEXa-I eligibility criteria are: (i) hematoma volume ≥ 0·5 to ≤ 60 mL; (ii) last NOAC intake ≤15 hours prior to randomization, or documented anticoagulant activity >100 ng/mL if last NOAC intake >15 hours prior to randomization or unknown time of last intake; (iii) time from onset <6 hours prior to the baseline imaging scan; (iv) NIHSS score ≤35 and (v) GCS score ≥7.

Supplementary Table S1. Participating sites and investigators in alphabetical order

Site	Investigators
Cantonal Hospital **Aarau**	Sandra Clarke, Martina Falcao, Krassen Nedeltchev
University Hospital **Basel**	Nikolaos S Avramiotis, Leo H Bonati, Gian Marco De Marchis, Tolga Dittrich, Stefan T Engelter, Urs Fisch, Joachim Fladt, Henrik Gensicke, Lisa Hert, Grzegorz M Karwacki, Philippe A Lyrer, Marina Maurer, Alexandros A Polymeris, Sabine Schaedelin, Iris Schneider, Sebastian Thilemann, Christopher Traenka, Benjamin Wagner, Martina Wiegert, Annaelle Zietz
University Hospital **Bern**	Morin Beyeler, Urs Fischer, Martina Goeldlin, Marianne Kormann, Basel Maamari, Liselotte McEvoy, Thomas R Meinel, Madlaina Müller, David J Seiffge, Jan Vynckier
Cantonal Hospital **Lucerne**	non-recruiting site
Cantonal Hospital **St. Gallen**	Elena Ardila, Ingrid Feuerstein, Claudine Gantenbein, Georg Kägi, Anna Müller, Vjosa Seljmani, Jochen Vehoff, Benedikt Wagner
Klinic Hirslanden **Zurich**	Bettina Anders, Roland Backhaus, Filip Barinka, Asterios Paliantonis, Nils Peters, Shadi Taheri, Dennis Thumm
University Hospital **Zurich**	Jannie van Duinen, Lita Von Bieberstein, Susanne Wegener

Supplementary Table S2. Major thromboembolic events and re-initiation of oral anticoagulation in the intention-to-treat population

S2A. Number of participants with major thromboembolic events and re-initiation of oral anticoagulation and their timing according to treatment allocation and concomitant treatment with 4-factor prothrombin complex concentrate

	**All (N=63)**	**Tranexamic acid (N=32)**		**Placebo (N=31)**	
		**4fPCC (N=22)**	**No 4fPCC (N=10)**	**4fPCC (N=19)**	**No 4fPCC (N=12)**
Participants with major thromboembolic events within 90 days	6 (9.5%)	2 (9·1%)	2 (20%)	2 (10.5%)	0 (0%)
Participants with re-initiation of oral anticoagulation within 90 days	14 (22.2%)	6 (27·3%)	3 (30%)	2 (10.5%)	3 (25%)
Time to re-initiation, days	56 (31-64·5)	32 (28·5-59·5)	56 (55·5-62)	47 (38·3-54·8)	57 (47·5-61)
Major thromboembolic events before re-initiation*	6 (9.5%)	2 (9.1%)	2 (20%)	2 (10·5%)	0 (0%)
Major thromboembolic events after re-initiation	0 (0%)	0 (0%)	0 (0%)	0 (0%)	0 (0%)

S2B. Details of participants with major thromboembolic events

**Age and sex**	**Major thromboembolic event**	**Time from ICH onset to thromboembolic event**	**Study treatment allocation and concomitant 4fPCC treatment**	**Time from ICH onset to re-initiation of oral anticoagulation**
75 years, female	pulmonary embolism	16 days	tranexamic acid and 4fPCC	75 days
87 years, female	myocardial infarction, pulmonary embolism, Leriche syndrome (diagnosed simultaneously)	29 days	tranexamic acid and 4fPCC	no re-initiation
82 years, female	ischemic stroke	17 days	placebo and 4fPCC	no re-initiation
82 years, male	ischemic stroke	14 days	placebo and 4fPCC	no re-initiation
72 years, male	myocardial infarction	21 days	tranexamic acid, no 4fPCC	68 days
79 years, male	deep vein thrombosis	15 days	tranexamic acid, no 4fPCC	55 days

Data in 2A are median (IQR) or n (%). 4fPCC = 4-factor prothrombin complex concentrate; ICH = intracerebral hemorrhage. *Including major thromboembolic events that occurred in participants who were never restarted on oral anticoagulation.

Supplementary Table S3. Baseline characteristics of the per-protocol population

	Tranexamic acid (N=29)	Placebo (N=30)
**Age**, years	82 (75-87)	80·5 (78-84)
**Sex**		
Male	15 (52%)	20 (67%)
Female	14 (48%)	10 (33%)
**National Institutes of Health Stroke Scale score**	14 (8-18)	10·5 (5-19)
**Glasgow Coma Scale score**	14 (12-14)	14 (12-15)
**Pre-stroke modified Rankin Scale score**	0 (0-3)	1 (0-2)
**NOAC type**		
Apixaban	4 (14%)	7 (23%)
Dabigatran	0 (0%)	0 (0%)
Edoxaban	1 (3%)	2 (7%)
Rivaroxaban	24 (83%)	21 (70%)
**NOAC plasma level available on admission**	25 (86%)	27 (90%)
plasma level, ng/ml	116 (63-205)	138 (70-254·8)
**Indication for NOAC**		
atrial fibrillation	27 (93%)	24 (80%)
venous thromboembolism	2 (7%)	3 (10%)
embolic stroke of undetermined source	0 (0%)	2 (7%)
Unclear	0 (0%)	1 (3%)
**History of***		
hypertension	25 (86%)	28 (93%)
diabetes mellitus	7 (24%)	5 (17%)
ischemic stroke	3 (10%)	9 (30%)
intracerebral hemorrhage	0 (0%)	2 (7%)
**Blood pressure on admission**, mmHg		
systolic	163 (145-177)	170.5 (157-182)
diastolic	97 (78-110)	99 (86-114)
**Concomitant acute treatment with**		
NOAC-specific reversal agent	0 (0%)	0 (0%)
4-factor prothrombin complex concentrate	20 (69%)	18 (60%)
4-factor prothrombin complex concentrate dose, IU	1,900 (1,500-2,750)	1,500 (1,000-2,000)
**Time metrics**, hours		
symptom onset to baseline imaging	2·2 (1·4-3·6)	2·3 (1·5-5·2)
symptom onset to study treatment	5·2 (2·8-10·1)	4 (2·9-7·2)
last intake of NOAC to study treatment	15·2 (6·0-22·8)	10·9 (6·3-24·6)
**Intracerebral hemorrhage location**		
non-lobar (including infratentorial)	19 (66%)	24 (80%)
lobar (including mixed lobar and non-lobar)	10 (34%)	6 (20%)
**Baseline hematoma volume**, ml[†]	10·4 (4·8-35·6)	11·3 (5·7-22·4)
**Intraventricular hemorrhage extension**	8 (28%)	9 (30%)
**CT angiography done†**	27 (93%)	25 (83%)
spot sign positive	3 (11%)	2 (8%)
spot sign negative	24 (89%)	23 (92%)

Data are median (IQR) or n (%). NOAC = non-vitamin K antagonist oral anticoagulant. *The presence of the following comorbidities was unknown, and they were considered absent: diabetes (one participant), history of ischemic stroke (four), history of intracerebral hemorrhage (one). [†]One site used MRI, instead of CT, for baseline and follow-up imaging in three participants.

Supplementary Table S4. Primary and secondary outcomes in the per-protocol population

	**Tranexamic acid (N=29)**	**Placebo (N=30)**	**Effect size (95% CI)**	**p value**
**Primary Outcome**				
Hematoma expansion*	11 (38%)	14 (47%)	0·66 (0·23 to 1·91)†	0·45
**Secondary Outcomes**				
Symptomatic hematoma expansion*	8 (28%)	9 (30%)	0·86 (0·27 to 2·70)†	0·80
Absolute hematoma volume change, ml*	3·5 (0·5-7·7)	1·9 (0·3-8·7)	0·16 (-3·37 to 3·70)†	0·93
Ordinal modified Rankin Scale Score at 90 days			1·04 (0.41 to 2·68)†	0·93
0	0 (0%)	0 (0%)		
1	2 (7%)	3 (10%)		
2	3 (10%)	3 (10%)		
3	3 (10%)	3 (10%)		
4	6 (21%)	7 (23%)		
5	3 (10%)	2 (7%)		
6	12 (41%)	12 (40%)		
Modified Rankin Scale Score 0 – 4 at 90 days	14 (48%)	16 (53%)	0·86 (0·30 to 2·45)†	0·77
Modified Rankin Scale Score 0 – 3 at 90 days	8 (28%)	9 (30%)	0·91 (0·29 to 2·85)†	0·88
In-hospital death	5 (17%)	6 (20%)	0·83 (0·22 to 3·08)†	0·78
Death within 90 days	12 (41%)	12 (40%)	0·99 (0·33 to 2·93)†	0·99
Major thromboembolic events within 90 days	4 (14%)	2 (7%)	2·01 (0·39 to 10·33)	0·40
Ischemic stroke	0	2		
Myocardial infarction	2‡	0		
Deep vein thrombosis / pulmonary embolism	3‡	0		
Neurosurgical intervention up to day 2	1 (3%)	0 (0%)	··	··

Data are n (%) or median (IQR). Effect sizes are reported as adjusted median difference for the absolute hematoma volume change, common odds ratio for ordinal mRS and odds ratios for all other outcomes, except for neurosurgical intervention (presented only descriptively due to small number of events). *Missing follow-up imaging for two participants (one tranexamic acid, one placebo; not included in the absolute hematoma volume change analysis, but included in the hematoma expansion analyses assuming worst outcome); One site used MRI, instead of CT, for baseline and follow-up imaging in three participants. †Adjusted for baseline hematoma volume. ‡One patient assigned to tranexamic acid had both myocardial infarction and pulmonary embolism diagnosed simultaneously 29 days after intracerebral hemorrhage onset.

Supplementary Figure S1. Recruitment of evaluable randomized participants stratified by recruiting site over time

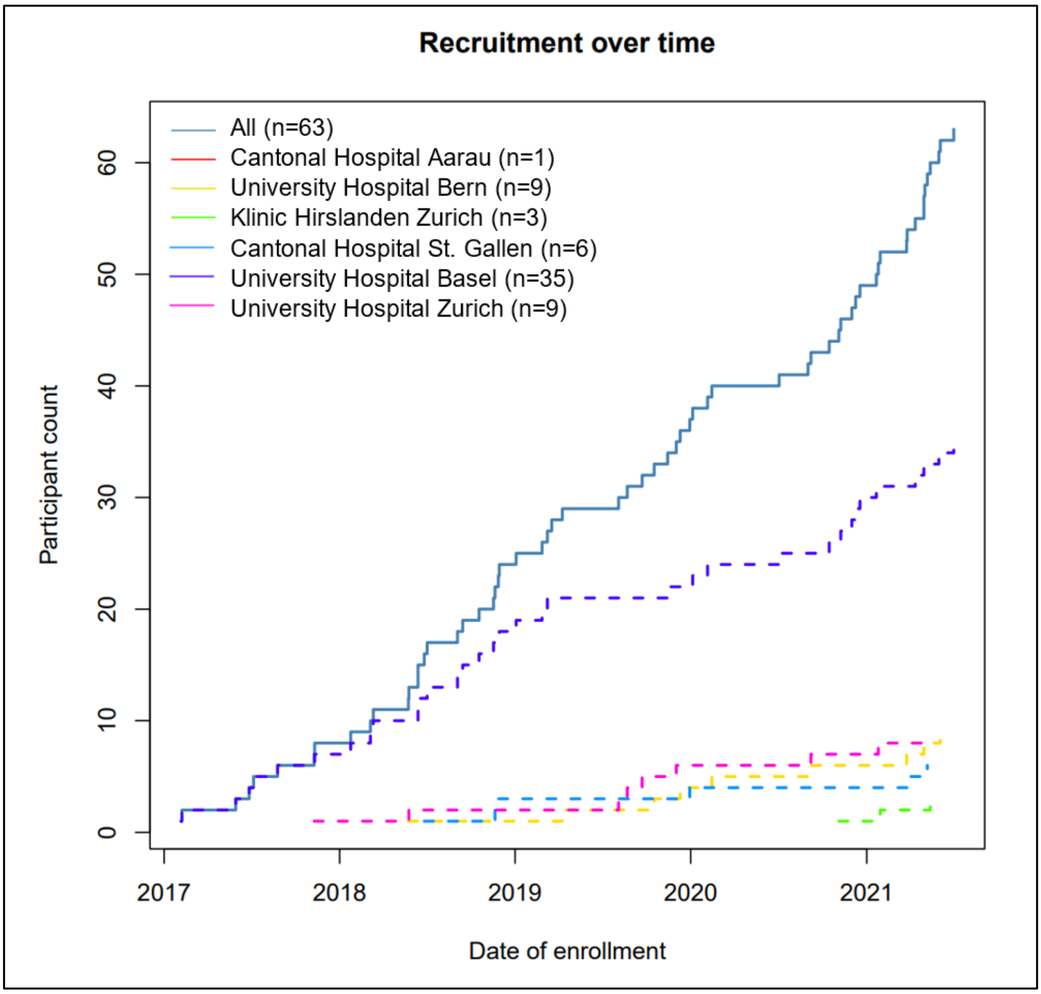

Supplementary Figure S2. Shift plot of modified Rankin Scale score at 90 days in the intention-to-treat population

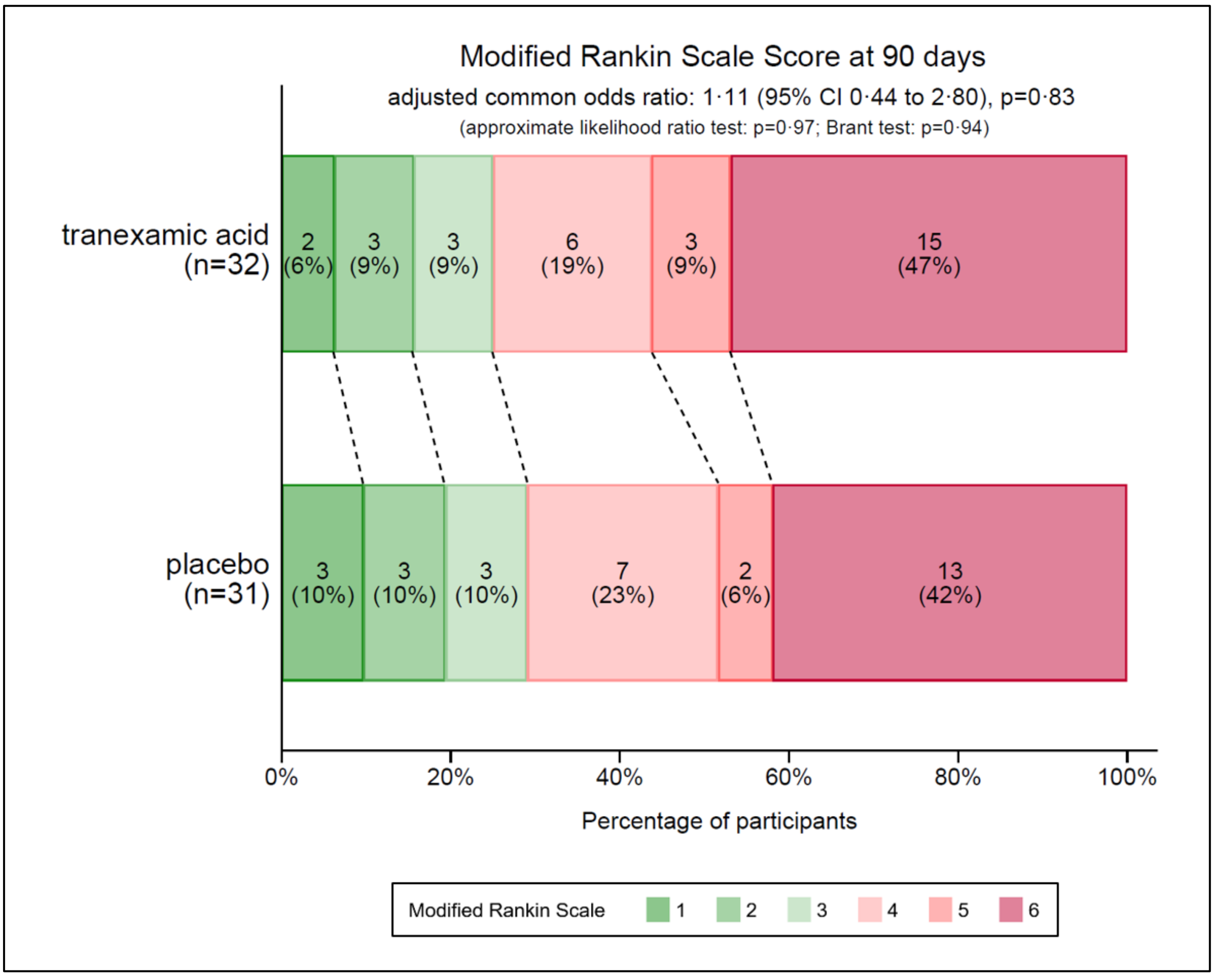

1 = no significant disability, 2 = slight disability, 3 = moderate disability, 4 = moderately severe disability, 5 = severe disability, 6 = dead

Supplementary Figure S3. Histogram of the absolute hematoma volume change from baseline to follow-up imaging in the intention to treat population

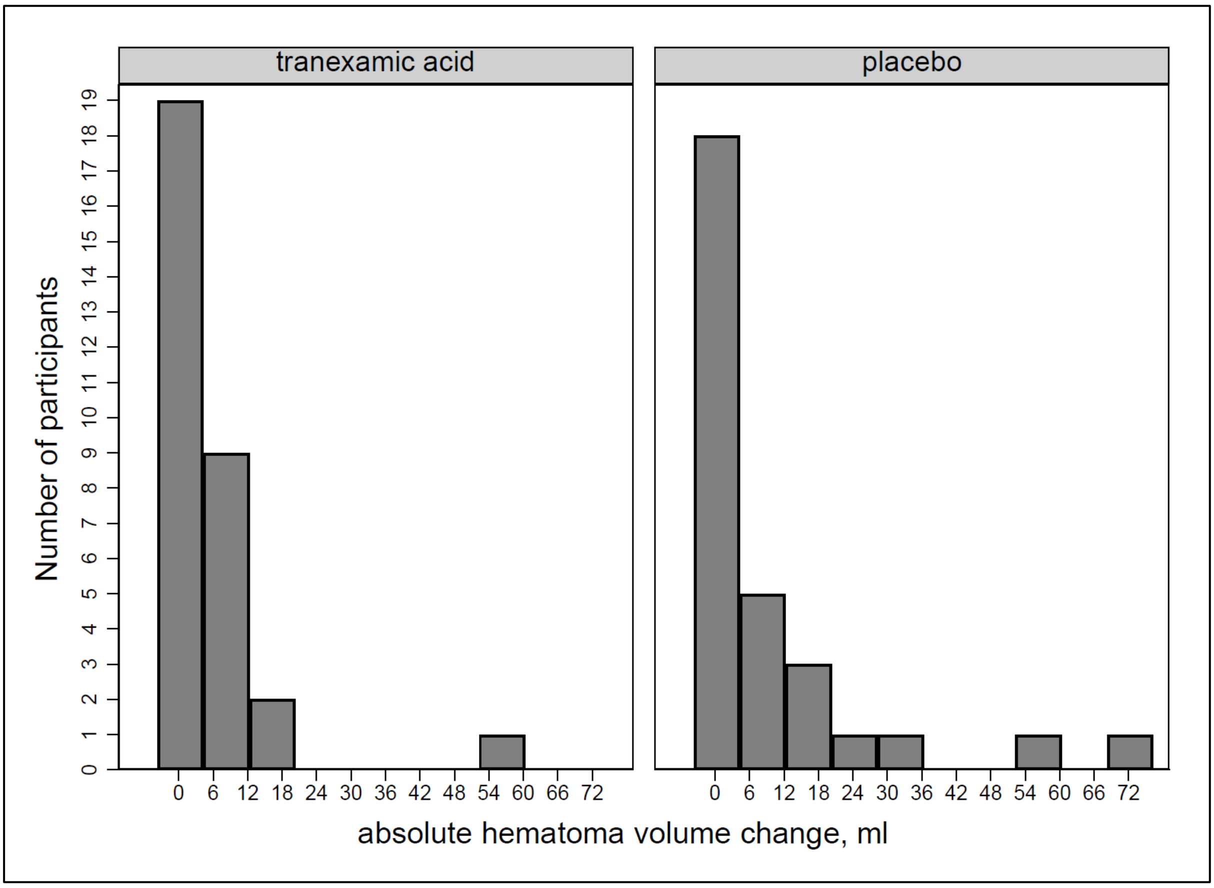

There were fewer "big expanders" in the tranexamic acid than the placebo arm. Not included are two participants without follow-up imaging (one assigned to tranexamic and one to placebo).

Supplementary Figure S4. Primary outcome by subgroups according to onset-to-treatment time in the intention-to-treat population (exploratory analysis)

Subgroup	Participant number	Odds ratio (95% CI)	Interaction p-value
Onset to treatment time, h			0.063
≤7.5	42	0.31 (0.08, 1.25)	
>7.5	21	4.99 (0.42, 59.97)	
Onset to treatment time, h			0.024
≤6	37	0.23 (0.05, 1.00)	
>6	26	3.85 (0.56, 26.55)	
Onset to treatment time, h			0.180
≤4.5	28	0.27 (0.05, 1.55)	
>4.5	35	1.23 (0.29, 5.24)	
Onset to treatment time, h			0.184
≤3	21	0.22 (0.03, 1.81)	
>3	42	0.97 (0.27, 3.52)	

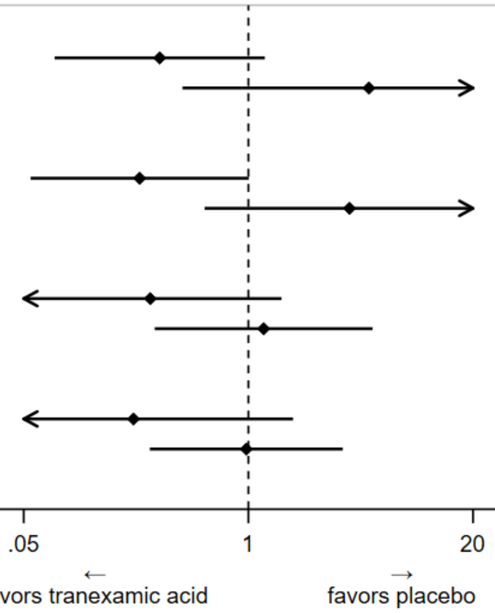

All odds ratio estimates are adjusted for baseline hematoma volume and were derived in each subgroup analysis from two separate models (one for each category of the binary subgroup, fitted after excluding participants of the other category).

Supplementary Figure S5. Boxplot of absolute hematoma volume change from baseline to follow-up imaging in the per-protocol population

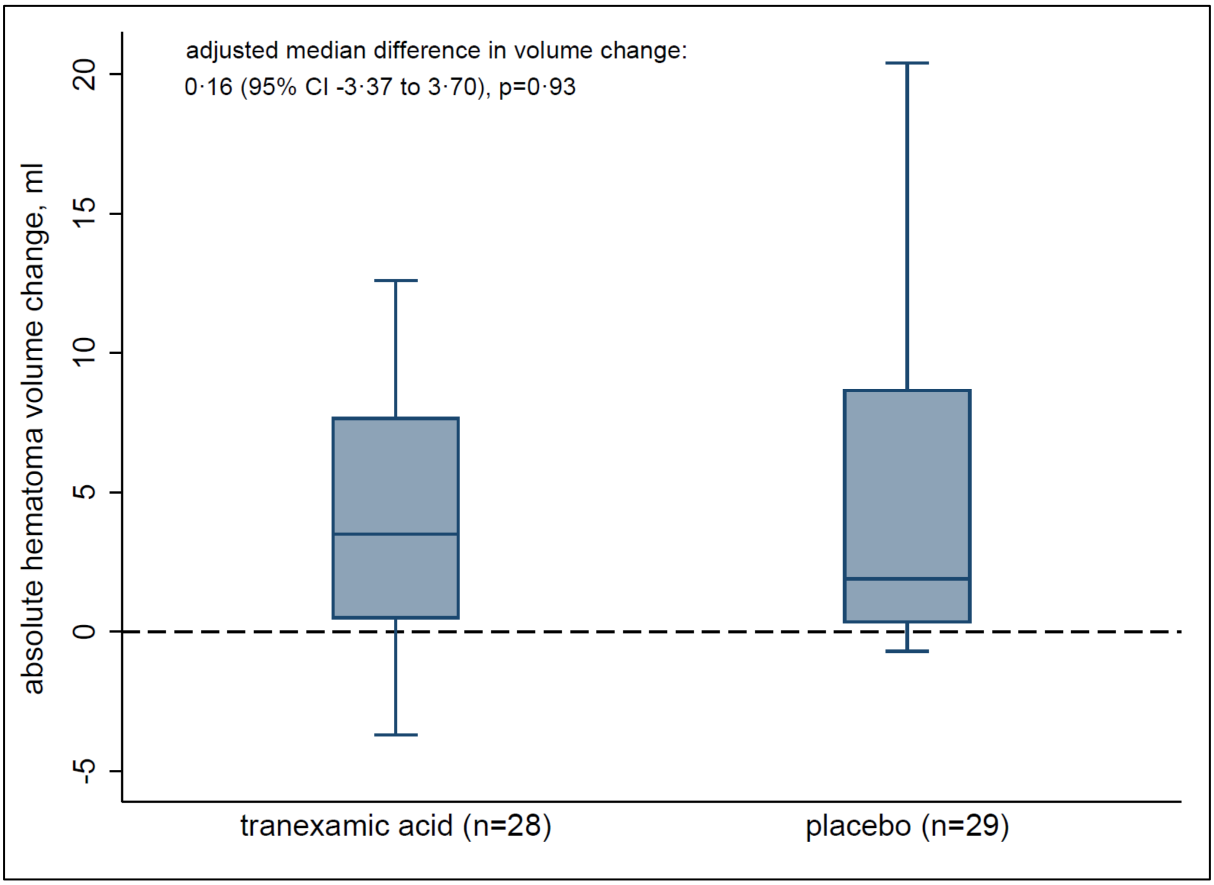

Boxes show median and IQR, the whiskers show the full range of values excluding outliers over 1·5 times the IQR beyond the IQR limits. Not included are two participants without follow-up imaging (one assigned to tranexamic and one to placebo).

Supplementary Figure S6. Shift plot of modified Rankin Scale score at 90 days in the per-protocol population

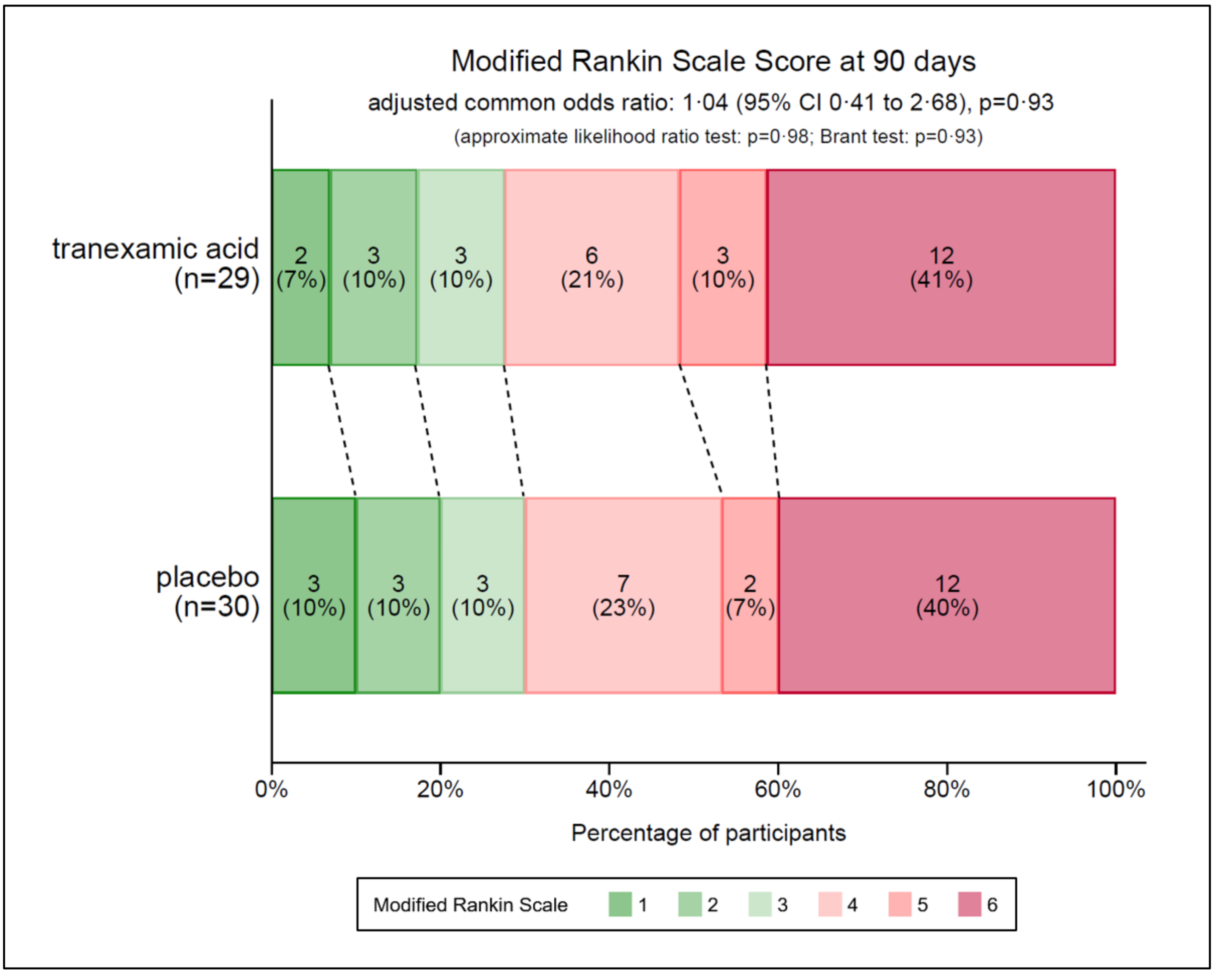

1 = no significant disability, 2 = slight disability, 3 = moderate disability, 4 = moderately severe disability, 5 = severe disability, 6 = dead

Supplementary Figure S7. Primary outcome by subgroups in the per-protocol population

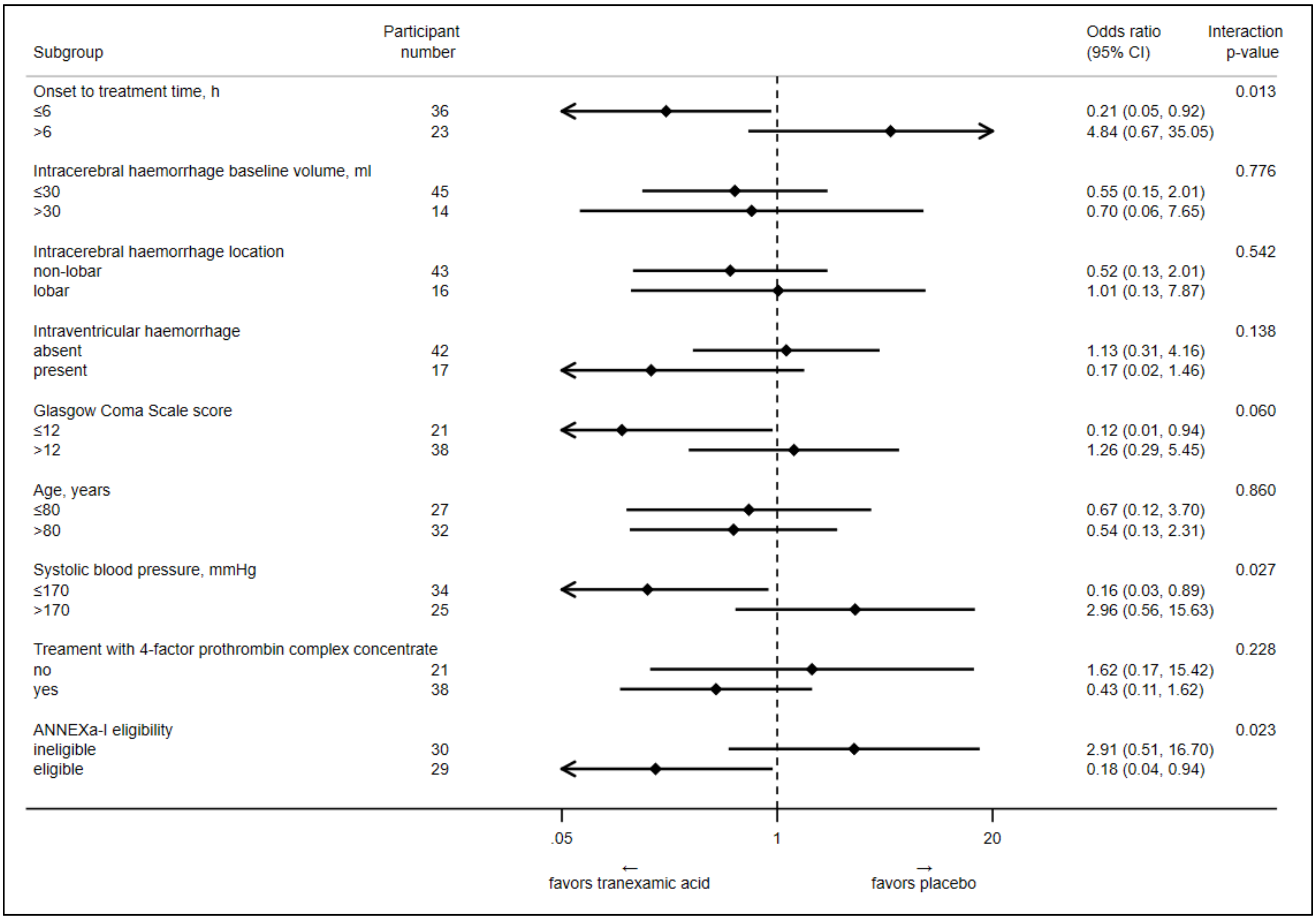

All subgroups were predefined except for systolic blood pressure, concomitant treatment with 4-factor prothrombin complex concentrate, and ANNEXa-I eligibility. All odds ratio estimates are adjusted for baseline hematoma volume and were derived in each subgroup analysis from two separate models (one for each category of the binary subgroup, fitted after excluding participants of the other category).

Supplementary Figure S8. Sample size calculation assuming the observed effect

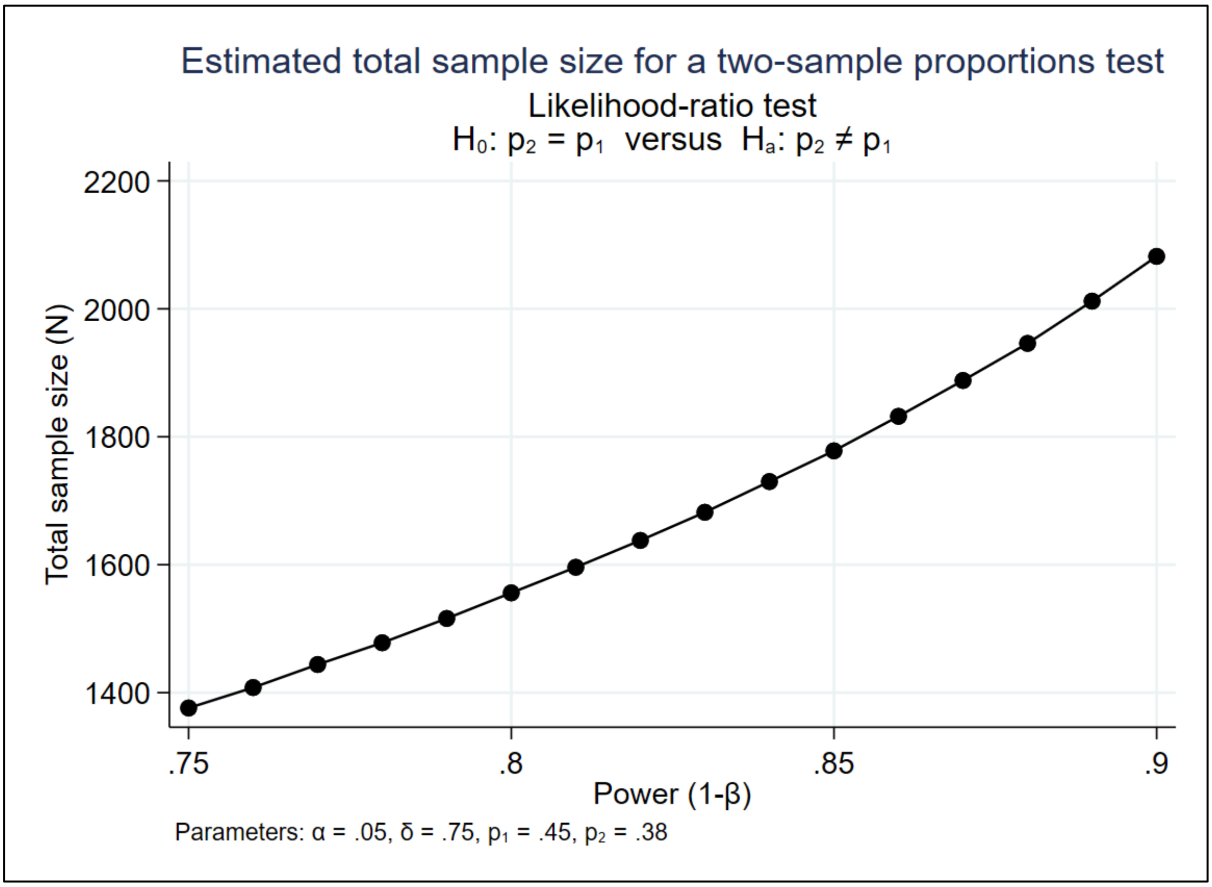

To demonstrate statistical significance for the observed effect (hematoma expansion rate of 38% in the intervention arm versus 45% in the control arm) with an allocation ratio of 1:1 and power of 80% at a two-sided alpha of 5%, a total of 1,556 participants (778 per arm) would have been required in TICH-NOAC.
